# Stimulus–response bindings in priming

**DOI:** 10.1016/j.tics.2014.03.004

**Published:** 2014-07

**Authors:** Richard N. Henson, Doris Eckstein, Florian Waszak, Christian Frings, Aidan J. Horner

**Affiliations:** 1 MRC Cognition and Brain Sciences Unit, Cambridge, UK; 2 Institut für Psychologie, Universität Bern, Bern, Switzerland; 3 Center for Cognition, Learning, and Memory, Universität Bern, Bern, Switzerland; 4 Institut Neurosciences Cognition, Université Paris Descartes, Paris, France; 5 CNRS Laboratoire Psychologie de la Perception UMR 8242, Université Paris Descartes, Paris, France; 6 Allgemeine Psychologie und Methodenlehre, Universtät Trier, Trier, Germany; 7 Institute of Cognitive Neuroscience, University College London, London, UK; 8 Institute of Neurology, University College London, London, UK

**Keywords:** S–R bindings, repetition suppression, automaticity, masked priming, subliminal priming, negative priming

## Abstract

People can rapidly form arbitrary associations between stimuli and the responses they make in the presence of those stimuli. Such stimulus–response (S–R) bindings, when retrieved, affect the way that people respond to the same, or related, stimuli. Only recently, however, has the flexibility and ubiquity of these S–R bindings been appreciated, particularly in the context of priming paradigms. This is important for the many cognitive theories that appeal to evidence from priming. It is also important for the control of action generally. An S–R binding is more than a gradually learned association between a specific stimulus and a specific response; instead, it captures the full, context-dependent behavioral potential of a stimulus.

## Highlights

• S–R bindings are more flexible and pervasive than previously thought.
• S–R bindings can simultaneously encode multiple stimulus and response representations.
• S–R bindings can be encoded or retrieved in the absence of attention or awareness.
• S–R bindings complicate interpretations of priming, but are interesting in their own right.
• S–R bindings enable rapid yet context-dependent behaviors.

## Introduction

Our daily lives involve interacting with a large number of stimuli. Many of these stimuli occur not only once, but recur at different timescales. We therefore need to learn rapidly how to process these recurring stimuli, without necessarily intentionally recalling prior experiences with those stimuli. One example of this rapid learning is priming, in which a change in the mental processing of a stimulus is normally measured by an overt behavioral response cued by that stimulus (Box 1). Priming is often interpreted as facilitation of one or more of the computations, or ‘component processes’ [1], that are necessary to generate that response. In a typical laboratory experiment, for example, participants might press one of two buttons depending on whether they judge a visually presented object to be living or nonliving, for which priming would be apparent if their average reaction time (RT) for this judgment is shorter for repeated than for initial presentations of the objects. In this example, the component processes that are facilitated might include perceptual identification of the object depicted (perceptual priming) and/or retrieval of semantic information about that object (conceptual priming).

**Figure 1:**
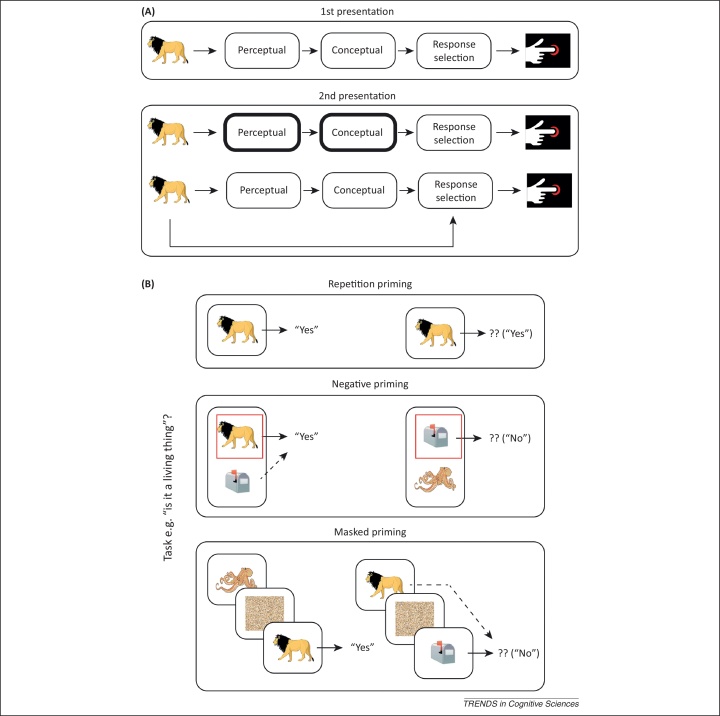
Schematic of component processes, stimulus–response (S–R) bindings, and priming paradigms. **(A)** When someone is asked to make a decision about a stimulus (e.g., whether the object depicted by an image is living or nonliving), several component processes are required to, for example, identify perceptually the object (here, a lion) and retrieve conceptual information about it (that a lion is a living entity) (top row). When that stimulus is presented a second time, the reaction time (RT) to make the same judgment is normally faster, a phenomenon called priming. This could reflect facilitation of one or more of the component processes engaged on initial presentation (second row) or it could reflect retrieval of an S–R binding that encodes the stimulus and response made on the initial presentation, without needing to re-engage the original component processes (third row). **(B)** The three main types of priming paradigm considered here are repetition priming (top row), negative priming (middle row), and masked priming (bottom row). The initial presentation is shown on the left and the repeat presentation on the right. In the case of negative priming, the red square indicates the target stimulus to which participants attend to determine their response (other concurrent stimuli are distractors). In the masked priming case, the prime is often presented for less than 50 ms and followed by a backward mask (illustrated by a square of pixel noise here) to render it subliminal. The broken lines indicate potential encoding or retrieval of an S–R binding.

However, it has long been suspected that priming can also result from directly associating, or binding, a stimulus and response. If these S–R bindings are retrieved when the stimulus is repeated, the response can be produced without necessarily recapitulating the component processes that were initially used to generate that response (Figure 1A). Evidence for S–R bindings has been found in all major priming paradigms (Box 1 and Figure 1B): repetition priming [2], negative priming [3], and masked priming [4].

The study of S–R bindings is important for two reasons. First, the presence of S–R bindings potentially confounds interpretation of many priming effects; for example, whether unattended items are truly inhibited [5,6] or whether semantic representations can really be accessed unconsciously [4,7]. More importantly, S–R bindings are now recognized to play a vital role in the control of action; a role that goes beyond the mere acceleration of stimulus-driven action. For example, research reviewed below indicates that S–R bindings encode information at multiple levels of abstraction, furnishing considerable flexibility and context sensitivity in their deployment. However, despite the recent interest in S–R bindings, many crucial questions remain unanswered.

## Ubiquity and flexibility of S–R bindings

**Figure 2:**
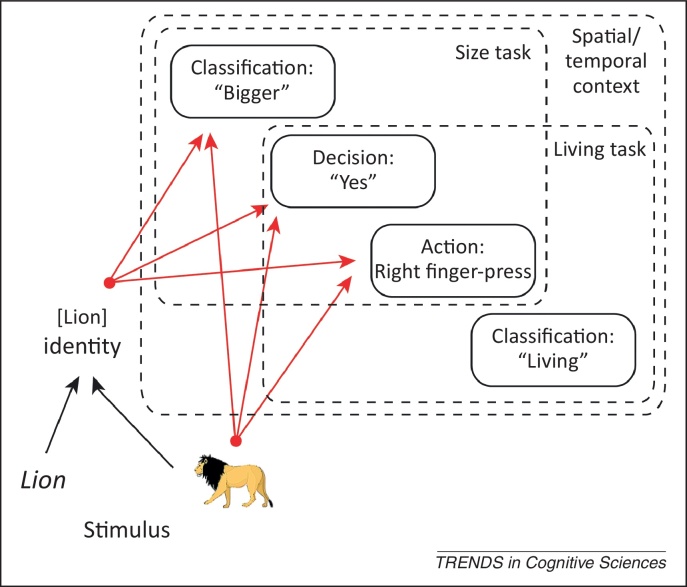
Possible stimulus–response (S–R) binding. Schematic of a possible structured S–R binding formed by giving a response to a picture of a lion during a binary ‘bigger than shoebox?’ categorization task, where red lines indicate bindings. Stimulus representations include a visual image of the picture and a more abstract representation of the identity of that stimulus, such that if the word ‘lion’ is later presented, it can also cue responses via the bindings between the identity representation and response representations. Response representations include a specific motor action (e.g., right index finger depression), a binary decision (e.g., ‘yes’) and a particular classification (e.g., ‘bigger’ in the size task). This means that retrieval of an action or decision can influence responses even if the task is changed; for example, to an ‘is the object living?’ categorization instead (as shown). Similarly, retrieval of a decision can influence responses even if the effector (action) is changed and retrieval of a classification can influence responses even if the task (and hence decision and action) is reversed (e.g., to a ‘smaller than shoebox?’ task). Retrieval of the S–R binding may also be mediated by the spatial/temporal context (e.g., laboratory setting).

Although the concept of S–R bindings is not new (Box 2), a recent resurgence in interest has been driven by evidence for their greater prevalence and flexibility than previously thought. For example, S–R bindings are far from being simple associations between a specific stimulus and specific response; rather, they appear to be structured bindings involving multiple levels of representation of responses, stimuli, and tasks (Figure 2). Moreover, these bindings do not need to be gradually learned; they can be formed from a single pairing of a stimulus and response.

## The nature of ‘R’

Despite evidence for effector-specific response codes [8], there is general agreement that responses can also be represented by their goal, rather than just specific motor programs [9,10]. Priming effects that survive a switch in effector between prime and probe are consistent with this claim (e.g., [11] in the case of negative priming).

However, priming that is invariant to a change in effector is not necessarily evidence for abstract response representations in S–R bindings, because the residual priming could reflect other factors, like facilitation of component processes (or inhibition of a stimulus representation in the case of [11]). To identify the level of response representation within an S–R binding, one must measure the difference in priming between a congruent condition (where the same response is given each time a stimulus is presented) and an incongruent condition (where the opposite response is given) or an unrelated condition (where a different type of response is given). In the case of repetition priming, for example, Horner and Henson [12] showed a reduction in priming when the response to a repeated stimulus switched from a vocal yes/no to a manual key press (relative to the congruent condition of a manual response to both prime and probe). This suggests that the specific motor action is indeed encoded in the S–R binding. However, priming from a vocal yes/no response to manual response was still greater than when the prime object was simply named. This suggests that S–R bindings additionally encode more abstract response representations, such as a yes/no decision (see also [13]).

Further research has suggested that S–R bindings can encode even more abstract response representations, such as the particular classification made (such as living versus nonliving) [14–18]. By changing the reference object during a relative-size judgment task (an experimental manipulation introduced by [19]), Horner and Henson [12] found greater repetition priming for objects that maintained the same bigger/smaller classification despite this reference change than for objects for which the classification changed. In the latter case, note that the yes/no decision and motor action were unchanged; only the classification label changed. These data suggest that S–R bindings can simultaneously encode at least three levels of response representation: action, decision, and classification (Figure 2). Furthermore, response representations like these have been shown to have independent [20,21] effects on behavior.

It has also been suggested that stimuli can be bound to nonspecific ‘stop codes’ that, when retrieved, inhibit responses in any ongoing task [22,23]. It has even been proposed that stimuli can be bound to attentional filters that have been previously applied to those stimuli [24]. This has been used to explain stimulus-specific congruency-proportion effects in Stroop tasks, where congruency effects are larger for stimuli that have been presented in contexts with a higher proportion of other congruent stimuli.

Multiple simultaneous levels of response representation potentially allow rapid execution of a specific action, as appropriate, for example, if the context (e.g., task) is unchanged, as well as allowing more flexible response options if the context changes. The downside of this flexibility for priming experiments is that simply changing the effector between prime and probe is not sufficient to ‘control for’ S–R bindings. Likewise, the presence of more specific response codes means that changing tasks between prime and probe is not a sufficient control either, if both tasks require a yes/no decision or the same motor action [25]. To properly investigate priming irrespective of the influence of S–R bindings, all levels of response representation must be controlled simultaneously [26].

## The nature of ‘S’

Similar questions relate to the nature of stimulus representations within S–R bindings. Priming, and its modulation by response congruency, has been shown to decrease with decreasing perceptual similarity between prime and probe [19,26,27], consistent with S–R bindings encoding relatively form-specific representations. However, response congruency effects have also been found despite switching from object pictures to written object names [26] or from object pictures to object sounds [28]. This again suggests that S–R bindings can encode multiple levels of stimulus representation, including at the abstract level of stimulus ‘identity’ (Figure 2).

Response congruency effects have been shown for semantically related stimuli [29,30]. This raises the question of whether bindings can be formed between responses and the features that comprise stimuli (see [31]). In the case of masked priming, for example, such ‘feature–response’ (F–R) bindings may explain priming from stimuli that occur only once in an experiment: so-called ‘novel’ primes [32]. This finding has been assumed to exclude S–R bindings (although see Box 2). If related stimuli have been seen (as probes) and paired with a response, such that features of those stimuli become bound with that response, later repetition of some of those features in a novel (but related) prime stimulus may be sufficient to retrieve the response and hence prime the subsequent probe. This hypothesis is consistent with priming by novel words that comprise parts of words seen previously as probes [33] and with claims that masked priming from novel stimuli occurs only when stimuli come from a small and tightly related stimulus set [32,34,35].

S–R bindings may also include representations of more than one concurrent stimulus. In negative priming paradigms, for example, there is evidence that the target and distractor stimulus also become bound together, independent of their binding to the response [23]. Such ‘S–S bindings’ seem to be determined by the principles of perceptual grouping [36]. There is also evidence of S–S bindings in associative priming tasks where the response requires comparing two or more concurrent stimuli [37]. Again, these data imply a more complex picture of S–R bindings than is normally conceived, including multiple levels of stimulus as well as response representation, bindings between stimulus features and responses, and bindings between multiple stimuli. This complexity affords yet further flexibility in, for example, allowing learned responses to be triggered not only by the same stimulus, but also by similar stimuli.

## Contextual bindings

Aspects of the concurrent context might also be bound with the stimulus and response. One example is the task set in which a stimulus is encountered. It has been shown that the typical ‘task-switch cost’, which reflects slower RTs for trials preceded by a different relative to same task, is increased if stimuli are repeated across the tasks [38]. This suggests that the repetition of a stimulus automatically retrieves the previous task set associated with that stimulus, which can interfere with any new task set (also see [14,39]). Importantly, Waszak and colleagues [17,40] argued that S–R bindings are more likely to be retrieved if they were compiled under a task set that remains active during the probe trial (given that a previous task set remains active for a certain time after a task switch: so-called ‘task-set inertia’ [41]). Task set-dependent retrieval clearly makes adaptive sense, in that one would not want all previous responses that have been associated with a stimulus constantly to compete with current behavioral goals (cf. ‘utilization’ behavior [42,43]). Other types of spatial or temporal context (e.g., laboratory setting) may also mediate S–R retrieval. Overlap in this level of context may explain why prior responses can still be cued by a repeated stimulus despite a switch in task [12], at least when specific response options are shared between the tasks (as in Figure 2).

More recently, a new line of research has explored how S–R bindings might be formed simply by verbal instruction [44–48]. For example, Wenke and collaborators [49] presented participants with a set of S–R mappings (e.g., N = left key, K = right key) for one task (Task A). Before attempting Task A, participants performed another task, Task B. Although the instructed mappings for Task A were irrelevant to Task B, they interfered with the performance of Task B when the stimuli in Task B overlapped with those instructed. Instruction-based S–R bindings might allow people quickly to implement any arbitrary S–R mapping and to use this mapping to guide behavior early in the learning of complex skills. However, the nature of instructed S–R mappings is not yet well known. One possibility is that instructions result in covert execution of the instructed mapping, with results similar to the overt application of the mapping.

## Role of attention and awareness at encoding and retrieval

Although attention and awareness are intimately related, one can be aware of a stimulus despite it not being the focus of attention, as in negative priming, or one can attend to a specific point in time and space but not be aware of a stimulus presented at that point, as in masked priming. To what extent are attention and awareness important for the encoding and/or retrieval of S–R bindings?

The negative priming paradigm has shown that attention is not necessary for encoding S–R bindings. For example, Rothermund *et al.* [3] presented strings of five letters (e.g., BFBFB), in which only the second and fourth were task relevant. They found the standard negative priming effect when the distractor letters (in the other positions) became task relevant (i.e., targets) in a subsequent trial and the correct response was incongruent with that given on the original trial, but positive priming when the response was congruent. Indeed, it may make adaptive sense to bind all stimuli to responses when encoding new experiences, because one does not always know which stimulus will be relevant in the future. Frings and colleagues [50] also found a response congruency effect when distractor stimuli were repeated as distractors, suggesting that attention is not necessary for retrieval of S–R bindings either (see also [51–53] for evidence from repetition priming). Nonetheless, negative priming experiments using other stimulus configurations [54,55] or longer lags between repetitions (A. Horner, PhD thesis, University of Cambridge, 2010) suggest that attention may sometimes be necessary. One possibility is that bindings initially occur between all stimuli, attended or unattended, in a short-lived ‘event file’ [31], but only the bindings to attended stimuli last longer.

Although attention appears to be necessary for masked priming [56,57], response congruency effects in masked priming suggest that awareness is not necessary for retrieving S–R bindings [58]. Eckstein and Henson [58], however, found no evidence of response congruency effects for masked primes that were never seen unmasked, suggesting that awareness is necessary for encoding such bindings. Although other studies have found main effects of priming from primes never seen unmasked [32,59–61], this residual priming could reflect unconscious facilitation of component processes [59,60] rather than subliminal encoding of S–R (or F–R) bindings. Again, to establish the role of a factor like awareness or attention in the encoding or retrieval of S–R bindings *per se*, one needs to find an interaction between that factor and response congruency.

## Interactions between S–R bindings and component processes in response selection

Several questions remain about the nature of S–R bindings and how they interact with other processes to determine behavior. For example, does each pairing of a stimulus and response produce a new S–R binding or progressively strengthen an association between an existing stimulus and response representation? Either possibility can explain why response congruency effects tend to increase with the number of stimulus–response pairings [4,12,62,63]. The finding that the standard deviation as well as the mean of RTs scales with the number of pairings has been used to argue for separate S–R bindings that race independently to produce the response [64]. However, when responses from these two routes are incongruent, extra time seems necessary to resolve this discrepancy, slowing RTs relative to unprimed trials [26], which suggests that retrieval of S–R bindings interacts with component processes during the final stages of response selection (Box 2).

**Figure 3:**
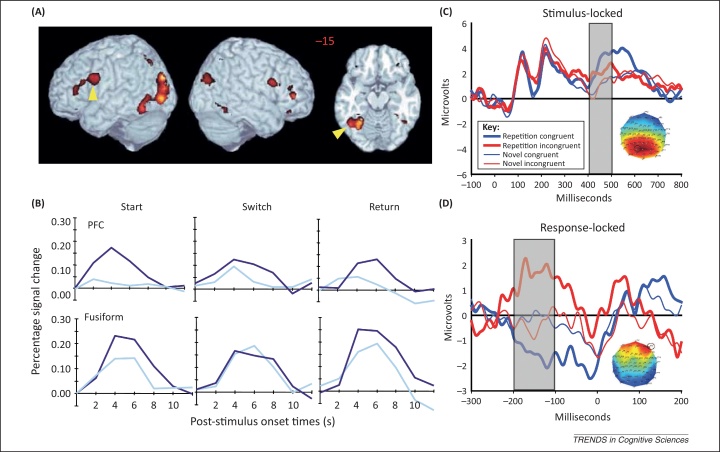
Neural correlates of stimulus–response (S–R) retrieval. **(A,B)** Data from the functional MRI (fMRI) study of Dobbins *et al.*[63] in which simply reversing the task in a repetition priming paradigm reduced repetition suppression (RS). In the start phase, participants judged whether visual objects were bigger than a shoebox; in the switch phase, the same objects (together with new, unprimed objects) were judged as to whether they were smaller than a shoebox (in the return phase, the original ‘bigger’ task was reinstated). The red patches in the three views of a canonical brain in (A) indicate regions showing smaller responses to primed than unprimed trials in the start phase (i.e., RS). The plots in (B) show the average fMRI evoked response to unprimed (dark blue) and primed (light blue) trials from two representative such regions: the prefrontal cortex (PFC) and the ventrotemporal cortex (fusiform). Note that RS in the fusiform is abolished when the task is reversed. Dobbins *et al.* suggested that the RS in the start phase reflected bypassing of component processes when S–R bindings are retrieved, whereas the lack of RS in the switch phase arises when S–R bindings are no longer used. Reproduced, with permission, from [63]. **(C,D)** Data from the event-related potential (ERP) study of Horner and Henson [80]. Participants performed the same size-judgment task as in Dobbins *et al.*, except that the referent object (e.g., a shoebox) was switched between prime and probe to render the previous response congruent or incongruent. (C) A time window (grey box) within a stimulus-locked ERP over parietal electrodes during which an effect of stimulus repetition was seen that was not modulated by whether the response was repeated or reversed between presentations (at least until later). (D) An effect over frontal electrodes showed a response congruency effect for primed (repeated) stimuli, but for not unprimed (novel) stimuli, a few hundred milliseconds before a key was pressed (i.e., response-locked). Whereas the stimulus-locked effect was hypothesized to reflect the facilitation of (perceptual) component processes, the response-locked effect was hypothesized to reflect decision processes that resolve the conflict when responses retrieved from S–R bindings and responses generated by component processes are incongruent. Reproduced, with permission, from [80].

The idea that potential responses retrieved from S–R bindings are vetted by a final stage of response selection affords an extra layer of cognitive control that is likely to be important. For example, in situations where strong top-down control is required, it may be possible to bias selection against the responses retrieved from S–R bindings and in favor of responses generated by component processes (for example, when accuracy is emphasized over speed). Thus, although retrieval of S–R bindings may not necessarily require awareness or attention, retrieval is not ‘automatic’, in the sense that it is modulated by contextual factors like task set (reviewed above), Gestalt mechanisms [36], semantic matching [51,65], and feedback [66]. The cognitive control of response selection then provides an extra level of flexibility, which means that, even when retrieved, S–R bindings do not necessarily dominate our behavior in an inflexible manner. However, the details of this response selection stage remain to be established, and would certainly benefit from computational modelling (Box 3) and possibly convergent evidence from neuroscientific data (Figure 3).

Although we have focused on RTs, incongruent S–R bindings may also lead to increased error rates [4,60,67–69]. In the case of negative priming, multinomial processing models have been used to analyze the probability of erroneous probe responses due to retrieval of the prime response [68,70]. If a stimulus from the prime episode is repeated in the probe, the probability of responding erroneously with the prime response is significantly increased compared with when no stimulus is repeated. Errors can therefore be understood as failures of component processes to overcome retrieval of S–R bindings in incongruent trials.

## Limitations of S–R bindings

Although we have emphasized the pervasiveness and flexibility of S–R bindings in priming, we should note that there are priming effects that cannot easily be explained by S–R bindings. One example is residual (positive) priming when all obvious levels of response code are reversed between prime and probe, or at least when there is no obvious overlap in response codes between prime and probe [12]. Such cases arise when tasks like naming or perceptual identification are performed, on the prime for example, together with a different (e.g., classification) task performed on the probe (or vice versa). In such cases, each stimulus would be associated with a unique response that is not repeated in the probe task so could not modulate priming. More generally, there is little doubt that prior processing of an intact visual object can modify subsequent perception of a degraded version (e.g., a binarized image, such as the famous Dalmatian dog [1]), such that the object is clearly seen when primed but not when unprimed, without any overt behavioral response being made. For further arguments about priming effects that are independent of S–R binding, see [12,60,71–73]. Moreover, researchers should be wary of automatically appealing to S–R bindings to explain priming unless there is direct evidence for their existence, such as modulations of the size of the priming effects by response congruency, as described above. Finally, because we have also raised the possibility of F–R and S–S bindings, it may seem that bindings can explain just about any aspect of human behavior (rendering them somewhat vacuous as explanatory concepts). However, we emphasize that S–R bindings are only assumed here to influence behavior in tasks where stimulus-cued responses overlap with previous responses to those stimuli; that is, in situations where there are after-effects of prior experience.

## Concluding remarks

Although the cognitive revolution dispensed with the behaviorist claim that all behavior can be understood in terms of S–R learning, such associations undoubtedly play a role in many of our behaviors. Importantly, S–R bindings are more than simple associations between a specific percept and motor act; they are complex, structured representations that simultaneously bind multiple levels of stimulus, response, and task representation. Furthermore, S–R bindings can, under certain experimental conditions, be encoded and retrieved in the absence of attention or awareness. This complexity and ubiquity make it difficult to control for S–R bindings when using priming to investigate other theoretical questions. Moreover, S–R bindings are no longer viewed only as a confound; they have become interesting in their own right (Box 4). Indeed, their ability to allow us to interact with our environment rapidly, yet also flexibly, suggests that they constitute a fundamental aspect of human cognition.

## Box 1 Major types of priming

Priming refers to a change in accuracy, bias, or reaction time to respond to a stimulus (‘probe’) owing to prior presentation of the same or a similar stimulus (‘prime’). It is indexed as the difference between the response to the prime and probe or between the probe and a comparable stimulus not presented before (‘unprimed’). There are at least three main priming paradigms, which differ in whether a response is made to: (i) the prime; and/or (ii) the probe (see Figure 1B in main text).

In repetition priming, a response is made to both prime and probe. The prime and probe typically occur in separate trials separated by multiple intervening trials (so that priming does not simply owe to repetition of the same response across successive trials; that is, ‘response priming’). Note that the stimulus may not be repeated in exactly the same physical form (e.g., it may switch from a written to a spoken word across trials).

In negative priming, the prime (and sometimes the probe) is a distractor; that is, it is not the focus of attention, such that the response is generated instead by a different, concurrent stimulus. Responses are typically slowed when the prime is then presented as a probe in a subsequent trial (although note that priming is not always ‘negative’ in the sense of a slowing; positive priming can occur if the response previously paired with the prime is congruent with that required to the probe).

In masked priming, the prime is masked to render it subliminal, such that no response can be made to it. The subsequent supraliminal probe typically occurs within a few hundred milliseconds (i.e., within the same trial). The S–R binding that is potentially retrieved by the masked prime then usually comes from previous presentations of the same stimulus as a probe in other trials.

These paradigms differ in other ways too. For example, repetition priming can survive lags of many intervening trials and sometimes last days, whereas negative priming typically occurs only across successive trials and masked priming rarely lasts beyond one trial. There are also other priming paradigms (e.g., semantic/affective priming, where prime and probe are different stimuli but semantically or affectively related), but here we focus on paradigms in which S–R bindings have been shown to play a dominant role, and these normally involve repeating a stimulus to cue retrieval of an S–R binding (although see text about possible F–R bindings).

## Box 2 S–R theories

There are many theories relating to S–R bindings; we focus on one example pertinent to each of the three priming paradigms considered here (Box 1).

Logan proposed an ‘instance theory’ [64] of automaticity that has been mainly applied to repetition priming. Responses are determined by a race between an algorithmic route (comprising computations used to produce a response the first time a stimulus is encountered) and retrieval of any relevant S–R bindings (with a separate such ‘instance’ stored each time a response is given to a stimulus). Assuming that all S–R bindings race independently, RTs will decrease with the number of instances according to a power law, with the mean and standard deviation having the same exponent. A later version of the theory [74] included two additional decision rules (a counter and a random-walk model) that prioritize response accuracy at the cost of increased RTs in conflict situations. With this extension, instance theory can also potentially explain negative priming.

Hommel [31] hypothesized the existence of ‘episodic records’. These encode features of stimuli and responses, with each record binding only two features (which can be from different stimuli), although the same feature can occur in multiple records. Hommel's theory was developed primarily to explain ‘carry-over’ costs from one trial to the next (such as negative priming), rather than longer-lived facilitatory effects seen in repetition priming paradigms.

Kunde *et al.* [75] proposed an ‘action trigger’ theory (see also [76]), which is most often applied to masked priming. The basic idea is that repeated pairings of a stimulus and response establish a trigger that releases an action when a related stimulus reappears. The generalization to related stimuli comes from being able to specify the trigger conditions in broad terms. For example, evidence that the masked digit ‘3’ can prime a relative size judgment to a subsequent probe ‘4’, even if the 3 was not presented previously in the experiment [60], can be explained by a generic trigger of the sort ‘small numbers should produce a “no” response’.

Although these S–R theories have proved helpful, each needs further development to explain the precise role of attention and awareness during encoding and retrieval of S–R bindings (see text) and their interactions with other component processes. Moreover, few if any theories specify neural mechanisms that can be compared with recent brain data (Box 3).

## Box 3 S–R bindings in the brain

Another reason for the resurgence of interest in S–R bindings concerns recent neuroimaging and neuropsychological data. In particular, the phenomenon of ‘repetition suppression’ has been assumed to reflect the facilitation of component processes and therefore used to map neural representations in different brain regions [77,78]. An influential functional MRI (fMRI) study by Dobbins *et al.* [63], however, suggested that repetition suppression in ventrotemporal regions (associated with visual object perception) reflects instead the bypassing of such processes owing to retrieval of S–R bindings (see Figure 3A in main text). Although later studies suggested that S–R bindings cannot explain all repetition suppression in perceptual regions, retrieval of S–R bindings clearly has important effects on neuroimaging data [15,79]. Recent work has focused on relating the effects of response congruency in prefrontal cortex to the integration of: (i) responses retrieved from S–R bindings; and (ii) responses generated from component processes [15,80].

Effects of response congruency have been found in response-locked event-related potentials (ERPs) over frontal electrodes a few hundred milliseconds before the response occurs [15,80] (see Figure 3D in main text). ERPs locked to stimulus onset, by contrast, appear less affected by response congruency, suggesting that stimulus repetition effects on these ERPs may reflect facilitation of component processes. Stimulus repetition also modulates 5–15-Hz power in ventrotemporal regions as measured by magnetoencephalography (MEG) [81] and increases the synchrony of this oscillatory activity between prefrontal and ventrotemporal regions [82] (cf. [83]). Although these MEG studies did not manipulate response congruency, they raise the potential importance of changes in communication between brain regions [81]. The importance of such interactions was reinforced by a study showing that transcranial magnetic stimulation of the prefrontal cortex abolished repetition suppression in ventrotemporal regions [84].

Although amnesic patients with damage to the medial temporal lobes (MTLs) have long been claimed to show intact priming [85], Schnyer *et al.* [86] found no effect of response congruency in such patients. This is consistent with hippocampal lesions in animals, which typically disrupt learning of arbitrary visuomotor associations [87]. One tentative possibility is that S–R bindings are stored in the MTLs (even if not necessarily in a conscious manner) that, when retrieved, interact in the prefrontal cortex with responses generated by component processes in the ventrotemporal cortex. Finally, there are also relevant data from single-cell recording in animals, and neurally plausible computational models are clearly vital to integrate all these types of data [88,89].

## Box 4 Outstanding questions

• How do S–R bindings and component processes interact; for example, to affect decision processes that select the final behavioral response?
• How are S–R bindings structured? For example, does a single binding contain multiple stimulus and response representations (as in Figure 2 in main text) or does each stimulus and response representation form a separate ‘binary’ S–R binding? Under what conditions do complex versus specific S–R bindings form? Are simple S–R bindings formed from one learning episode, whereas more complex S–R bindings require more, and more varied, learning episodes?
• How are S–R bindings retrieved? If they contain multiple stimulus and response representations (as in Figure 2 in main text), how do matching and mismatching stimulus representations affect the probability of retrieving a binding? If there are multiple, binary S–R bindings, how do they compete for retrieval – is a single winning binding retrieved or do multiple bindings feed into a decision process?
• What are the limits of stimulus and response representations in S–R bindings? For example, are individual stimulus features bound to responses? How are contexts like task set represented: as part of the same binding or as some kind of index that selects (activates) those S–R bindings that are currently relevant?
• Do different types of S–R binding have different lifetimes (potentially accounting for differences across repetition, negative, and masked priming paradigms) and how long do they last relative to facilitation of component processes?
• How exactly do attention and awareness modulate the encoding and retrieval of S–R bindings?
• How many previous claims for component processes (e.g., in fMRI) reflect S–R bindings instead?
• Which brain regions enable S–R bindings and by what neural mechanisms do they interact with component processes?
